# Transfusion related babesiosis in a non-endemic region (Western New York)

**DOI:** 10.1093/omcr/omae133

**Published:** 2024-11-20

**Authors:** Joseph Aladeen, Pranav Wadhawan, Reshub Pendyala, Bikram Bajwa, Rajinder P Bajwa

**Affiliations:** Pharmacy Department, Niagara Falls Memorial Medical Center, 621 10th street, Niagara Falls, NY 14301, United States; Student, Ridley College, 2 Ridley Road, St. Catharines, ON L2R 7C3, Canada; Student, Nichols School, 1250 Amherst Street, Buffalo, NY 14216, United States; Student, Nichols School, 1250 Amherst Street, Buffalo, NY 14216, United States; Infectious Disease Division, Department of Medicine, Niagara Falls Memorial Medical Center, 621 10th street, Niagara Falls, NY 14301, United States

**Keywords:** Babesia, blood transfusion, hemolytic anemia

## Abstract

Babesiosis, an emerging tick-borne zoonosis caused by parasites of the genus Babesia, is typically transmitted via the black-legged tick. Occasionally, *Babesia* can also be transmitted through red blood cell transfusion. In this report, we present a case of babesiosis resulting from a red blood cell transfusion in an area where the pathogen is not endemic. The patient presented with a high-grade fever and hemolytic anemia. This case underscores the critical importance of recognizing Babesia as a cause of hemolysis and emphasizes the necessity of implementing universal screening of blood products for Babesia. Enhanced vigilance in blood safety protocols is imperative to prevent transfusion-associated babesiosis.

## Case report

A 20-year-old male with a significant medical history of sickle cell disease and frequent pain crises presented to the emergency department of a hospital in Niagara County, NY, complaining of lower back pain, frontal headache, and generalized weakness. Upon presentation, the patient exhibited a temperature of 39.5 degrees Celsius, a hemoglobin level of 7.7 gm/dl, and a white blood cell count of 13 100 cells/mm3. Notably, the patient denied recent travel or insect bites and reported no injection drug use. He disclosed having received transfusions with 5 units of packed red blood cells during an inpatient admission at a nearby hospital approximately 1 month prior to presentation. Additionally, on examination, a deep perineal abscess was noted. Consequently, the patient was admitted to the medical floor for treatment and stabilization.

During the admission, the patient underwent several units of packed red blood cell transfusions, received broad-spectrum antibiotics, and underwent incision and drainage of the perineal abscess. A subsequent culture of the abscess intraoperatively revealed the growth of methicillin-resistant *Staphylococcus aureus*, while blood and urine cultures showed no growth. Despite appropriate treatment and the perineal wound healing, the patient’s fevers persisted over the first six days following admission. Furthermore, he exhibited hemolytic anemia, with a total bilirubin level of 7.3 mg/dl and a hemoglobin level of 6.9 gm/dl, necessitating additional transfusions of packed red blood cells.

**Table:** 

Labs	
WBC	13.1
Hgb	6.9
Hct	18.3
Plt	275
RBC	2.19
BUN	6
Creatinine	0.56
T Bili	7.3
D Bili	4.0
ALP	75
LDH	1794

WBC-White blood cell, Hgb-Hemoglobin, Hct-hematocrit, Plt-Platelet, RBC-Red Blood Cell, BUN-Blood Urea Nitrogen, T Billi-Total Bilirubin, D Billi-Direct Billirubin, ALP-Alkaline Phosphtase, LDH-Lactate Dehydrogenase.

On the sixth day of admission, an infectious disease consultation was sought. The differential diagnosis was narrowed down after a negative direct Coombs test and a peripheral blood smear showing no schistocytes, effectively excluding autoimmune hemolysis and microangiopathic hemolytic anemia. Subsequently, a peripheral blood smear was ordered to assess for parasitemia, revealing rare red blood cell inclusions. The smear was then sent to the New York State lab for further identification. Empirical therapy for both malaria and babesiosis was initiated with atovaquone-proguanil and azithromycin. Babesia microti DNA was subsequently identified via PCR, with parasitemia measuring less than 0.1%. Therapy was streamlined to atovaquone and azithromycin accordingly.

Notably, the patient’s presentation occurred in March, which lies outside the typical peak tick season in the northeastern United States. Despite this, the patient denied any outdoor activities, recent travel to an endemic region, or recollection of tick bites. Repeat smears conducted after 5 and 9 days of treatment revealed persistent parasitemia of 1% and less than 0.1%, respectively. The patient’s fevers resolved after 7 days of treatment. Following improvement, the patient was discharged after 12 days of therapy, with treatment continuing for a total of 30 days with atovaquone and azithromycin. A subsequent smear after 28 days of therapy demonstrated no parasites.

Subsequently, the local health department and the blood donation service that supplied the patient’s blood products were notified of the probable case of transfusion-associated babesiosis. An investigation was launched by these organizations to identify the infected donor. At the time of writing this article, the infected donor has yet to be identified.

## Discussion

Babesiosis, an emerging tick-borne zoonotic illness endemic to the northeastern and upper Midwestern United States, is caused by intracellular protozoal parasites from the genus *Babesia* [1]. While several species of *Babesia* are known to cause disease in humans, the majority of cases in the United States are attributed to *Babesia microti*. The black-legged tick (*Ixodes scapularis*) serves as the primary vector for transmission, although congenital transmission, blood transfusion, and organ transplantation have also been reported, albeit rarely [1]. Despite its rarity, babesiosis represents the most frequently reported transfusion-related parasitic infection and is among the most common transfusion-transmitted infections [2]. From 1979 to 2009, a total of 162 cases of transfusion-transmitted babesiosis were reported in the United States, with 159 caused by *Babesia microti* [3].

In immunocompetent patients, babesiosis commonly manifests as mild or asymptomatic infections. However, in older or immunocompromised individuals, severe infection can occur, with a mortality rate as high as 20% [1].

The case presented above underscores the imperative for enhanced screening of donated blood products for *Babesia microti* species, even in regions where this pathogen is not endemic. The expanding range of *I. scapularis* over the past decade has coincided with an increased incidence of tick-borne infections, including babesiosis. Correspondingly, there has been a rise in reported cases of babesiosis overall, particularly those linked to transfusion [4–6].

While many otherwise healthy patients with babesiosis remain asymptomatic, parasitemia can persist for extended periods, up to two years, making it challenging to identify infected blood samples solely through patient history questionnaires [2]. This underscores the necessity for reliable tests to detect the organism in donated blood.

In March 2018, the FDA approved the *Babesia microti* Arrayed Fluorescent Immunoassay and the *Babesia microti* Nucleic Acid Test for screening donated blood and blood products for *Babesia* [2, 7]. However, the discontinuation of these tests in November 2018 prompted the FDA to approve the Procleix Babesia Assay and the cobas Babesia test in 2019 for similar purposes [2, 8].

In May 2019, the FDA issued recommendations for reducing the risk of transfusion-transmitted babesiosis, advocating for year-round screening of all donated blood products from states where natural transmission of babesiosis occurs [2]. This includes the use of licensed nucleic acid tests for screening and the implementation of quarantine and notification protocols for individuals testing positive.

Clinicians need to maintain a high level of awareness regarding babesiosis as a possible cause in patients who develop fever and hemolytic anemia after receiving a blood transfusion, even in regions where the disease is not typically endemic. The prompt adoption of FDA blood product screening recommendations, and potential expansion to all 50 states, holds promise for significantly mitigating the risk of future cases of transfusion-related babesiosis.
